# Entwicklung von Befragungsinstrumenten zur Erfassung von Walkability und Bikeability für das Präventionsindikatorensystem der Länder

**DOI:** 10.1007/s00103-025-04163-w

**Published:** 2025-12-02

**Authors:** Clara Tristram, Jonas D. Finger, Sophie John, Kristin Manz, Anne K. Reimers

**Affiliations:** 1 Department für Sportwissenschaft und Sport, Friedrich-Alexander-Universität Erlangen-Nürnberg, Gebbertstraße 123b, 91058 Erlangen, Deutschland; 2 Senatsverwaltung für Wissenschaft, Gesundheit und Pflege, Berlin, Deutschland; 3 Abteilung Epidemiologie und Gesundheitsmonitoring, Robert-Koch-Institut, Berlin, Deutschland

**Keywords:** Mobilitätsverhalten, Stadtplanung, Gesundheitsförderung, Indikatorenentwicklung, Infrastrukturqualität, Mobility behavior, Urban planning, Health promotion, Indicator development, Infrastructure quality

## Abstract

**Hintergrund:**

Bewegung ist eine wichtige verhaltensbezogene Gesundheitsdeterminante, die durch die Gestaltung einer bewegungsfreundlichen Umwelt beeinflusst werden kann. Ziel der Arbeit war die Entwicklung von Befragungsinstrumenten zur Erfassung von Walkability und Bikeability für die Präventionsberichterstattung der Länder.

**Methodik:**

Im Rahmen des Verbundprojekts „Monitoring von körperlicher Aktivität und Bewegungsförderung - Entwicklung von Indikatoren für das Präventionsindikatorensystem der Länder (KAB-Mon)“ (01.05.2023–30.04.2026) wurden zunächst mittels Literaturrecherche bestehende Befragungsinstrumente zur Messung von Walkability und Bikeability identifiziert, auf deren Grundlage Entwürfe für eigene Instrumente entwickelt wurden. Nach einer Bewertung durch 9 bzw. 7 Expertinnen und Experten anhand zuvor festgelegter Kriterien im Rahmen einer Online-Befragung wurden die Instrumente überarbeitet. Anschließend erfolgten leitfadenbasierte Interviews mit 18 Teilnehmenden, in denen die Instrumente getestet und bewertet wurden. Die Ergebnisse der Online-Befragung wurden durch Mittelwertvergleiche analysiert, die kognitiven Interviews qualitativ ausgewertet.

**Ergebnisse:**

Die Befragung der Expertinnen und Experten führte zu einer Kürzung des Walkability-Instruments von 16 auf 13 Items sowie des Bikeability-Instruments von 19 auf 14 Items. Nach dem kognitiven Test wurden beide Instrumente um eine fünfte Antwortoption („weiß nicht/keine Angabe“) ergänzt.

**Diskussion:**

Die im Rahmen des Projekts entwickelten Instrumente bieten einen fundierten wissenschaftlichen Ansatz zur subjektiven Erfassung von Walkability und Bikeability im deutschsprachigen Raum.

**Zusatzmaterial online:**

Zusätzliche Informationen sind in der Online-Version dieses Artikels (10.1007/s00103-025-04163-w) enthalten.

## Abstract (en)

**Background:**

Physical activity is an important behavioural determinant of health that can be influenced by designing a physical activity-friendly environment. The aim of this work was to develop survey instruments for walkability and bikeability for the prevention reporting of the federal states.

**Methods:**

As part of the project „Monitoring physical activity and promoting exercise - developing indicators for the federal states’ prevention indicator system in Germany (KAB-Mon)“ (01/05/2023–30/04/2026), the development and review of two instruments designed to subjectively assess the two prevention indicators walkability and bikeability were carried out in three steps. In Step 1, a systematic literature review was conducted. In Step 2, an expert survey followed in which 9 and 7 experts from 6 disciplines evaluated the items of both instruments using the specific criteria in an online survey. Step 3 involved cognitive testing through guided interviews with a total of 18 participants. The expert survey was analysed using mean comparisons, while the cognitive interviews were evaluated descriptively.

**Results:**

Based on the expert feedback, the walkability instrument was shortened from 16 to 13 items, and the bikeability instrument from 19 to 14 items. Following the cognitive testing, both instruments were supplemented with a fifth response option (“don’t know/no answer”).

**Discussion:**

The instruments developed through this project provide a solid scientific approach for the subjective assessment of walkability and bikeability in the German-speaking context.

## Hintergrund

Ungesunde Verhaltensweisen wie Rauchen, körperliche Inaktivität, einseitige Ernährung oder Alkohol- und Drogenkonsum haben negative Auswirkungen auf die Gesundheit und belasten das öffentliche Gesundheitssystem [1, 2]. Dabei kann vielen dieser verhaltensbezogenen Gesundheitsrisiken durch die Veränderung von Lebenswelt- und Kontextfaktoren vorgebeugt werden. Beispielsweise kann durch die Gestaltung einer bewegungsfreundlichen Umwelt die körperliche Aktivität gesteigert werden [3]. Um gesundheitsschädlichen Verhaltensweisen entgegensteuern zu können, müssen deren Einflussfaktoren zunächst erkannt, definiert und bewertet werden, damit anschließend entsprechende Präventionsmaßnahmen ergriffen werden können.

Das 2015 in Kraft getretene Präventionsgesetz (PrävG) hat den Bedarf aufgezeigt, die Präventionsberichterstattung der Länder mithilfe sogenannter Präventionsindikatoren der Länder zu vereinheitlichen [4]. Die Ausarbeitung von 2 bisher noch nicht entwickelten Präventionsindikatoren (Walkability und Bikeability) aus Themenfeld 5 „Bewegung/körperliche Aktivität“ des Präventionsindikatorensystems der Länder ist eines der Ziele des Verbundprojekts „Monitoring von körperlicher Aktivität und Bewegungsförderung - Entwicklung von Indikatoren für das Präventionsindikatorensystem der Länder (KAB-Mon)“, welches im Rahmen des Förderaufrufs des Bundesministeriums für Gesundheit (BMG) „Strukturelle Stärkung und Weiterentwicklung des Öffentlichen Gesundheitsdienstes (ÖGD)“ entstand (OEGD-Struktur-008). Nach ihrer Ausarbeitung können die beiden Indikatoren „Walkability“ (dt. häufig: „Fußgängerfreundlichkeit“) und „Bikeability“ (dt. häufig: „Fahrradfreundlichkeit“) zum Monitoring von lebensweltbezogenen Determinanten von Bewegung und körperlicher Aktivität genutzt werden.

Walkability und Bikeability umfassen nicht nur die Begehbarkeit bzw. Befahrbarkeit einer Umgebung, sondern die gesamte Bewegungsfreundlichkeit von Straßenzügen, Wohnvierteln, Stadtteilen und urbanen Räumen [5]. Umgebungsfaktoren können objektiv gemessen werden, beispielsweise durch spezielle Prüfungen (Audits) der Infrastruktur oder durch geografische Informationssysteme (GIS-Daten). Die subjektive Wahrnehmung, wie fußgänger- oder fahrradfreundlich die Umgebung ist, kann mittels Interviews oder Fragebögen erhoben werden [6, 7]. Während Fragebögen im englischsprachigen Raum bereits vorliegen [7–9] und teilweise auch im deutschsprachigen Raum Anwendung finden [10, 11], sind diese meist aufgrund ihrer hohen Anzahl an Items zu zeitaufwendig und somit für den Einsatz in bevölkerungsbezogenen Gesundheitssurveys eher ungeeignet.

Im Rahmen des Projekts sollen daher 2 kürzere Befragungsinstrumente entwickelt werden, mit denen die subjektive Wahrnehmung der Walkability bzw. der Bikeability erfasst werden kann. Im vorliegenden Beitrag werden der methodische Entwicklungsprozess sowie die erarbeiteten Instrumente beschrieben, aus deren Erhebungsdaten zukünftig Aussagen über die Walkability und Bikeability einer Umgebung für das Präventionsindikatorensystem der Länder abgeleitet werden können.

## Methodik

**Abb. 1:**
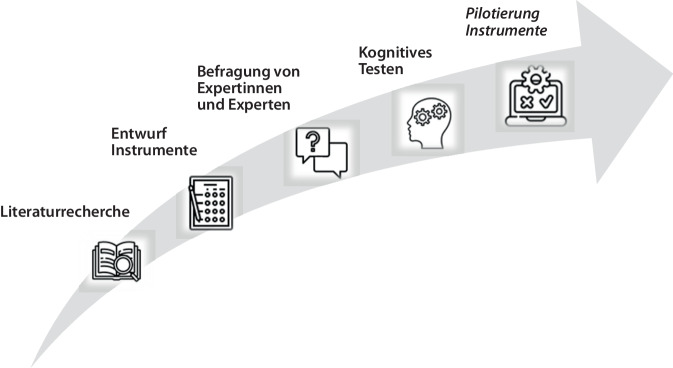
Methodisches Vorgehen bei der Entwicklung zweier Messinstrumente zur Erfassung der subjektiven Wahrnehmung von Walkability und Bikeability

Im Rahmen des KAB-Mon Projekts (01.05.2023–30.04.2026) erfolgte zunächst eine systematische Literaturrecherche, um Befragungsinstrumente für Walkability und Bikeability zu identifizieren. Die Ergebnisse der Literaturrecherche sind an anderer Stelle detailliert beschrieben [12]. Im Rahmen dieser Recherche konnten 3 bereits validierte Skalen identifiziert werden: NEWS‑G (Neighborhood Environment Walkability Scale; deutsche Version; [10]), ALPHA (Assessing Levels of Physical Activity; deutsche Version; [11]) und PANES (Physical Activity Neighborhood Environment Scale; englische Version; [13]). In Anlehnung an diese Befragungsinstrumente wurde ein erster Entwurf zweier Instrumente entwickelt, deren Items mithilfe einer Online-Befragung von Expertinnen und Experten bewertet wurden (Methoden siehe [14, 15]). In einem anschließenden kognitiven Testen mittels leitfadengestützter Interviews wurden die beiden Instrumente auf ihre Verständlichkeit, Einfachheit, den Gedankenprozess beim Ausfüllen, die Eignung der Antwortkategorien, soziale Erwünschtheit und die Sicherheit mit den Antworten getestet [16, 17]. Das methodische Vorgehen für die Entwicklung der beiden Befragungsinstrumente für Walkability und Bikeability ist in Abb. 1 überblicksartig dargestellt. Im Folgenden werden die Stichproben, die Datenerhebung und die Datenauswertung der Online-Befragung und des kognitiven Testens genauer beschrieben.

### Befragung von Expertinnen und Experten

Nachdem ein erster Entwurf der Befragungsinstrumente zur Erfassung von Walkability und Bikeability vorlag, wurden diese beiden Instrumente mithilfe einer Online-Expertenbefragung weiterentwickelt [15]. Das Ziel lag insbesondere in der Bewertung der Passung der einzelnen Items, der Überprüfung formeller Bedingungen (sprachliche Korrektheit, Verständlichkeit einzelner Begrifflichkeiten, Einheitlichkeit etc.) sowie der Kürzung der Instrumente, da diese in einem bevölkerungsbezogenen Gesundheitssurvey eingesetzt werden sollen.

#### Stichprobe.

Die Rekrutierung der Expertinnen und Experten erfolgte im Umfeld der Projektmitarbeitenden. Beim Sampling wurde darauf geachtet, dass die befragten Personen in unterschiedlichen Bereichen, wie der Wissenschaft (Sportwissenschaft, Verkehrswissenschaft, Expertinnen und Experten zur Methodik von Fragebogenerstellung), dem Verkehrs- und Mobilitätssektor oder der politischen Verwaltung, verortet waren. 30 Personen wurden kontaktiert.

#### Datenerhebung.

Die über Unipark laufende Befragung wurde mithilfe der Software Unipark (Tivian Global GmbH, Hamburg, DE https://www.unipark.com/) erstellt und vor dem Versenden an die Expertinnen und Experten innerhalb des KAB-Mon-Projektteams pilotiert. Der Fokus der Befragung lag auf der Bewertung der Items der beiden neu entwickelten Instrumente anhand modifizierter ZWERG-Kriterien (zentrale Bedeutsamkeit, Wirtschaftlichkeit, Einfachheit, Rechtzeitigkeit, Genauigkeit; [18]). Da diese Kriterien im Original der Überprüfung der Zielerreichung in Projekten zur Gesundheitsförderung und Prävention dienen, wurden sie für die Befragung im Rahmen der vorliegenden Arbeit so modifiziert, dass sie für die Bewertung der Items der beiden Instrumente zur Erfassung der subjektiven Wahrnehmung von Walkability und Bikeability passend waren. Die Modifikation beinhaltete vor allem das Entfernen der „Rechtzeitigkeit“ (R), da die rechtzeitige Fertigstellung der beiden Instrumente für die Expertinnen und Experten für die Bewertung der einzelnen Items nicht relevant erschien. Tab. 1 zeigt die ZWE(R)G-Kriterien, wie sie im Kontext der Befragung der Expertinnen und Experten verwendet wurden. Für jedes Item der beiden Instrumente sollte die Passung anhand der Kriterien bewertet werden. Hierfür standen den Befragten folgende Antwortmöglichkeiten zur Verfügung: 0 – gar nicht, 1 – eher nicht, 2 – teilweise, 3 – eher, 4 – vollständig, x – weiß nicht sowie ein Freitextfeld.

**Tab. 1:** ZWE(R)G-Kriterien (modifiziert nach [18]) zur Bewertung der Items der beiden Befragungsinstrumente zur Erfassung der subjektiven Wahrnehmung von Walkability und Bikeability

ZWE(R)G-Kriterium	Das Item …
Zentrale Bedeutsamkeit	… bildet präventionsrelevante Sachverhalte ab
	… ist wichtig für Steuerung und Koordinierung von Gesundheitsförderungspolitiken
	… hat eine Relevanz zur Verminderung sozial bedingter Ungleichheiten
Wirtschaftlichkeit	… ist notwendig zur Erfassung der Walkability bzw. Bikeability
Einfachheit	… ist leicht verständlich für Zielpersonen/Befragte
	… ist für die Fachöffentlichkeit und Politik leicht nachvollziehbar und interpretierbar
Genauigkeit	… ermöglicht eine zuverlässige Beurteilung (ist reliabel)
	… bildet einen Aspekt der Walkability bzw. Bikeability ab (ist valide)
	… bietet die Möglichkeit eines Vergleichs über Regionen und Zeitverläufe

Alle Expertinnen und Experten erhielten eine E‑Mail mit Informationen zur Teilnahme an der Online-Befragung, dem zugehörigen Link sowie den beiden Befragungsinstrumenten. Sie wurden über die Ziele und den Ablauf der Befragung informiert und über die geltenden Datenschutzrichtlinien (anonyme Datenverarbeitung und Protokollierung) aufgeklärt. Darüber hinaus stimmten alle Befragten zu Beginn der Online-Befragung ihrer Teilnahme zu. Das Ausfüllen der Online-Befragung dauerte ca. 15–20 min. Die Teilnehmenden erhielten keine Aufwandsentschädigung.

#### Datenauswertung.

Für die Auswertung der Befragung der Expertinnen und Experten wurden für jedes Item die Mittelwerte der 4 ZWE(R)G-Kriterien berechnet. Diese wurden im Anschluss addiert, sodass für jedes Item jeweils ein Wert für die 4 Bewertungskriterien vorlag (M_gesamt_). Auf Grundlage der Werte konnten Aussagen darüber getroffen werden, wie passend die Items aus Sicht der Befragten auf die Erfassung des jeweiligen Konstrukts (Walkability oder Bikeability) waren. Auf Basis der Ergebnisse wurden die beiden Instrumente gekürzt sowie sprachlich überarbeitet. Um das Ziel der Kürzung der Instrumente zu erreichen, wurde sich dafür entschieden, in dem Walkability-Instrument alle Items zu entfernen, deren M_gesamt_ < 2,50 aufwies. Für das Bikeability-Instrument wurde sich dafür entschieden, alle Items zu entfernen, deren M_gesamt_ < 2,70 aufwies, da die Bikeability-Items insgesamt einen höheren Mittelwert aufweisen als die des Walkability-Instruments.

### Kognitives Testen

Im Anschluss an die Befragung der Expertinnen und Experten folgte ein kognitives Testen der beiden überarbeiteten Instrumente mithilfe von Face-to-Face-Interviews [16, 17]. Das Ziel bestand in der Überprüfung der Verständlichkeit der beiden neu entwickelten Instrumente. Hierfür wurden insgesamt 18 leitfadengestützte kognitive Interviews geführt.

#### Stichprobe.

Um die Stichprobe für die kognitiven Interviews an die Bevölkerungsstruktur anzulehnen und eine diverse Zusammensetzung der Stichprobe zu gewährleisten, wurden die Befragten nach den folgenden Kriterien rekrutiert: (1) Alter, (2) Geschlecht, (3) Migrationshintergrund, (4) Bildungsabschluss sowie (5) Wohnlage. Die Rekrutierung der Teilnehmenden erfolgte im Umfeld der Projektmitarbeitenden.

#### Datenerhebung.

Nachdem die Teilnehmenden über die Ziele und den Ablauf der Interviews informiert und über die geltenden Datenschutzrichtlinien (anonyme Datenverarbeitung und Protokollierung) aufgeklärt worden waren und ihr schriftliches Einverständnis zur Teilnahme an dem Interview gegeben hatten, startete das Interview mit Tonaufnahme. Das Interview erfolgte mithilfe eines zuvor erarbeiteten Interviewleitfadens. Nach Abfrage ihrer soziodemografischen Angaben wurden die Interviewteilnehmenden gebeten, zuerst das Walkability-Instrument zu beantworten. Dabei wurde die Zeit gemessen, die sie für das Ausfüllen des Instruments benötigten. Darüber hinaus wurden Notizen zum Verhalten, wie beispielsweise längeres Zögern oder eine Veränderung der Reihenfolge beim Beantworten der Items, festgehalten. Nach dem Ausfüllen des Instruments wurden den Teilnehmenden spezifische Fragen zu einzelnen Items des Walkability-Instruments gestellt. Danach erfolgte das gleiche Vorgehen für das Bikeability-Instrument. Den Abschluss des Interviews bildeten allgemeine Fragen, die sich auf beide Befragungsinstrumente gleichermaßen bezogen. Der Schwerpunkt der spezifischen Fragen lag dabei auf der Überprüfung der Verständlichkeit, Einfachheit und Sicherheit der Antworten sowie darauf, den Gedankenprozess der Befragten bei der Beantwortung einzelner Items nachzuvollziehen. Der Fokus der allgemeinen Fragen hingegen richtete sich auf das Feedback der Befragten zu beiden Instrumenten gleichermaßen. In Anlehnung an T Lenzner et al. [16] und R Tourangeau et al. [19] wurde bei der Erstellung des Interviewleitfadens darauf geachtet, Fragen aus den folgenden Bereichen zu stellen: (1) Verständnisfragen (Comprehension), (2) Abruf- oder Erinnerungsfragen (Retrieval Probes), (3) Urteilsfragen (Judgment Probes) und (4) Antwortverhalten (Response Probes). Der Leitfaden ist dem Onlinematerial 1 zu entnehmen. Die Teilnahme am Interview erfolgte ohne Aufwandsentschädigung.

#### Datenauswertung.

Alle Interviews wurden auf Tonband aufgezeichnet und transkribiert. Die Transkription erfolgte mithilfe der Software f4transkript für Windows, Version 7.0.6 (lizenziert) und die Auswertung mithilfe von Windows Excel. Für die Auswertung wurde für jedes Interview zunächst ein Datenblatt erstellt, bei dem die Antworten der Teilnehmenden den einzelnen Fragen zugeordnet wurden. Anschließend wurden, getrennt nach dem Walkability- und dem Bikeability-Instrument, die Fragen innerhalb der 4 Bereiche analysiert. Dabei wurden speziell die Schwierigkeiten der Befragten, die mögliche Hinweise auf die Verständlichkeit einzelner Items gaben, analysiert. Die Auswertung erfolgte qualitativ in Anlehnung an die 4 Kategorien sowie das Coding-Schema von DeMaio und Landreth [20]. Das Datenblatt zur Auswertung ist dem Onlinematerial 2 zu entnehmen.

## Ergebnisse

### Befragung von Expertinnen und Experten

#### Merkmale der Stichprobe.

Von den insgesamt 30 kontaktierten Personen (15 Expertinnen und 15 Experten) beantworteten 7 die Online-Befragung vollständig. Zwei Personen beantworteten nur den ersten Teil der Befragung zum Walkability-Instrument. Daher wurden für das Walkability-Instrument Daten von 9 Expertinnen und Experten gewonnen (Response: 30 %), während für das Bikeability-Instrument nur von 7 Personen Ergebnisse zur Verfügung standen (Response: 23 %). Tab. 2 zeigt die Verteilung der Expertinnen und Experten nach ihrer Fachbereichszugehörigkeit.

**Tab. 2:** Übersicht über die Verteilung der Expertinnen und Experten nach Fachbereichszugehörigkeit

Fachbereich	Anzahl der Expertinnen und Experten
Politikfeld – Verkehr	1
Politikfeld – Sport	2
Wissenschaft – Sportwissenschaft	2
Wissenschaft – Prävention/Gesundheit	2
Wissenschaft – Verkehrswissenschaft	1
Wissenschaft – Geografie	1

#### Bewertung des Walkability-Instruments

Die Ergebnisse der Bewertung der Expertinnen und Experten der Walkability-Items anhand der ZWE(R)G-Kriterien sind in Tab. 3 festgehalten. Die Items 5, 9, 12, 13 und 15 weisen die niedrigsten Werte M_gesamt_ auf. Daher wurden die Items 5, 12, 13 und 15 aus dem Instrument entfernt. Für Item 13 ließ sich darüber hinaus ein Kommentar im Freitextfeld finden, in dem die befragte Person das Streichen dieses Items empfahl:

„Einige Items sind sehr deckungsgleich (‚fußgängerfreundlich‘, ‚familienfreundlich‘ etc.), obwohl ähnlich relevant … könnte man hier jeweils eins streichen“ (Antwort aus Freitextfeld).

**Tab. 3:** Bewertung der Walkability-Items durch die Expertinnen und Experten anhand der ZWE(R)G-Kriterien.

Nr.	Item	Bedeutsamkeit M (SD)	Wirtschaftlichkeit M (SD)	Einfachheit M (SD)	Genauigkeit M (SD)	Gesamt M (SD)
*1*	In meiner Wohnumgebung gibt es viele Einrichtungen des täglichen Bedarfs wie Geschäfte, Restaurants, Apotheken, Freizeiteinrichtungen und Schulen, die innerhalb von 10–15 min zu Fuß zu erreichen sind	3,50 (0,75)	3,50 (1,06)	3,37 (0,74)	2,75 (1,38)	3,28 (0,66)
*2*	In meiner Wohnumgebung gibt es eine gut ausgebaute Gehweginfrastruktur	4,0 (0)	3,62 (0,51)	3,0 (1,06)	3,12 (1,06)	3,25 (0,40)
*3*	In meiner Wohnumgebung wird den Fußgänger:innen genug Raum gegeben und die Gehwege sind ausreichend breit	3,12 (0,83)	3,25 (0,46)	2,75 (0,88)	2,37 (1,06)	2,87 (0,70)
*4*	In meiner Wohnumgebung sind die Gehwege in einem guten Zustand	3,0 (0,88)	3,25 (0,46)	3,50 (0,75)	3,12 (0,99)	3,21 (0,57)
*5*	In meiner Wohnumgebung gibt es Gehwege, die vom Verkehr getrennt sind^1^	3,25 (0,88)	2,12 (1,35)	2,0 (1,77)	2,37 (1,59)	2,43* (1,28)
*6*	In meiner Wohnumgebung kann ich Haltestellen des öffentlichen Nahverkehrs (Bus/Bahn) gut zu Fuß erreichen	3,75 (0,46)	3,37 (0,74)	3,87 (0,35)	3,75 (0,46)	3,68 (0,39)
*7*	In meiner Wohnumgebung fühle ich mich beim Zufußgehen vor Kriminalität sicher	3,0 (0,92)	2,87 (1,12)	3,12 (1,45)	2,37 (1,50)	2,84 (1,01)
*8*	In meiner Wohnumgebung gibt es viele verkehrsberuhigte Abschnitte wie 30er Zonen, Spielstraßen oder Fußgängerzonen	2,87 (1,55)	2,87 (1,24)	3,12 (0,99)	2,75 (0,88)	2,90 (1,07)
*9*	In meiner Wohnumgebung gibt es viele Zebrastreifen, Fußgängerampeln, Brücken oder Unterführungen^2^	2,50 (1,51)	2,37 (1,40)	2,50 (1,41)	1,75 (1,28)	2,28* (1,24)
*10*	In meiner Wohnumgebung fühle ich mich aufgrund der Verkehrssituation beim Zufußgehen sicher	2,87 (1,12)	2,50 (0,92)	2,87 (1,24)	2,37 (1,18)	2,65 (1,02)
*11*	Meine Wohnumgebung ist eine schöne Umgebung, um zu Fuß zu gehen	3,0 (0,75)	2,37 (0,74)	3,0 (0,92)	2,12 (0,64)	2,62 (0,59)
*12*	In meiner Wohnumgebung sind die Gehwege gut miteinander verbunden^1^	2,37 (0,51)	2,12 (1,24)	1,75 (1,03)	1,75 (1,16)	2,0* (0,8)
*13*	In meiner Wohnumgebung ist die Gehweginfrastruktur familienfreundlich^1^	2,50 (0,92)	2,14 (2,14)	1,85 (1,34)	1,71 (1,11)	2,03* (1,1)
*14*	In meiner Wohnumgebung gehen viele Menschen zu Fuß	2,57 (1,27)	2,62 (1,30)	3,37 (0,74)	2,87 (0,99)	2,96 (0,68)
*15*	In meiner Wohnumgebung ist die Gehweginfrastruktur in den letzten fünf Jahren deutlich verbessert worden^1^	2,42 (1,27)	2,12 (1,35)	2,50 (1,30)	2,37 (1,40)	2,46* (1,21)
*16*	Ich bewerte meine Wohnumgebung insgesamt als fußgängerfreundlich	3,75 (0,46)	3,37 (0,74)	3,62 (0,51)	3,25 (0,70)	3,50 (0,46)

*M_gesamt_ < 2,50

^1^Item entfernt

^2^Item nicht entfernt

Item 9 hingegen, welches mit M_gesamt_ = 2,28 deutlich unter dem festgelegten Niveau von 2,50 liegt, wurde nicht aus dem Instrument entfernt, da es für den Aspekt der „Verkehrssicherheit“, der einen zentralen Bestandteil des Konstrukts Walkability darstellt, als essenziell erachtet wurde [21]. Des Weiteren wurde ein neues Item zu „Barrierefreiheit“ eingefügt, da dies ebenfalls von einer befragten Person angeregt wurde:

„Es wird das breite Verständnis von Walkability abgefragt, bei dem das Thema Barrierefreiheit fehlt (abgesenkte Bordsteine, Blindenführungen auf dem Boden etc.). Treppen mit/ohne Rampen fehlen ebenfalls in der Abfrage“ (Antwort aus Freitextfeld).

#### Bewertung des Bikeability-Instruments

Die Ergebnisse der Bewertung der Expertinnen und Experten der Bikeability-Items anhand der ZWE(R)G-Kriterien sind in Tab. 4 zu finden. Die Items weisen insgesamt höhere Mittelwerte (M_gesamt_) auf als die Items des Walkability-Instruments. Weiterhin zeigen die Ergebnisse der Befragung, dass die Items 7, 12 und 18 einen geringeren M_gesamt_ als 2,70 aufweisen, weshalb diese aus dem Instrument entfernt wurden. Weiter wurden die Items 8 und 15 entfernt, obwohl sie mit M_gesamt_ = 3,08 (Item 8) bzw. M_gesamt_ = 2,89 (Item 15) über dem festgelegten Niveau von M_gesamt_ > 2,70 liegen. Begründet wurde dies zum einen mit dem Ziel der Einheitlichkeit zwischen den beiden Instrumenten (Walkability und Bikeability), da im Walkability-Instrument das Item 12 entfernt wurde, welches äquivalent zu Item 8 aus dem Bikeability-Instrument ist, und der Aspekt der Topografie, den das Item 15 adressiert, im Walkability-Instrument ebenfalls nicht zu finden ist. Zum anderen ist zu beachten, dass die Verfügbarkeit von E‑Bikes und Pedelecs das Transportverhalten von Personen heutzutage beeinflusst und dadurch die Topografie weniger als hinderlich für das Radfahren wahrgenommen wird [22].

**Tab. 4:** Bewertung der Bikeability-Items durch die Expertinnen und Experten anhand der ZWE(R)G-Kriterien.

Nr.	Item	Bedeutsamkeit M (SD)	Wirtschaftlichkeit M (SD)	Einfachheit M (SD)	Genauigkeit M (SD)	Gesamt M (SD)
*1*	In meiner Wohnumgebung gibt es viele Einrichtungen des täglichen Bedarfs wie Geschäfte, Restaurants, Apotheken, Freizeiteinrichtungen und Schulen, die innerhalb von 10–15 min mit dem Fahrrad zu erreichen sind	3,57 (0,78)	3,57 (1,13)	3,57 (0,53)	3,0 (1,41)	3,42 (0,71)
*2*	In meiner Wohnumgebung gibt es viele ausgewiesene Radstreifen und Radwege	3,85 (0,37)	3,85 (0,37)	3,71 (0,48)	3,28 (0,75)	3,67 (0,40)
*3*	In meiner Wohnumgebung gibt es viele ausgewiesene Radschnellwege und Fahrradstraßen	3,16 (1,60)	2,71 (1,70)	2,85 (0,69)	2,42 (1,51)	2,95 (1,12)
*4*	In meiner Wohnumgebung wird den Radfahrer:innen genug Raum gegeben und die Radwege sind ausreichend breit	3,57 (0,53)	3,42 (0,78)	3,14 (0,69)	2,85 (1,06)	3,25 (0,57)
*5*	In meiner Wohnumgebung sind die Radwege in einem guten Zustand	3,71 (0,48)	3,42 (0,53)	3,57 (0,78)	3,14 (1,06)	3,46 (0,41)
*6*	In meiner Wohnumgebung gibt es Radwege, die vom Verkehr getrennt sind	3,14 (0,69)	2,85 (1,21)	3,14 (0,69)	2,71 (1,11)	2,96 (0,78)
*7*	In meiner Wohnumgebung gibt es viele Fahrradampeln, -brücken und -unterführungen^1^	2,42 (1,27)	2,57 (1,27)	2,57 (1,13)	2,28 (1,49)	2,46* (1,15)
*8*	In meiner Wohnumgebung sind die Radwege gut miteinander verbunden^1^	3,50 (0,83)	3,16 (0,98)	3,16 (0,40)	2,50 (1,04)	3,08 (0,64)
*9*	In meiner Wohnumgebung kann ich Haltestellen des öffentlichen Nahverkehrs (Bus/Bahn) gut mit dem Fahrrad erreichen	3,0 (0,81)	3,0 (1,0)	3,28 (0,48)	2,85 (0,69)	3,03 (0,68)
*10*	Der öffentliche Nahverkehr (Bus/Bahn) in meiner Wohnumgebung bietet mir Gelegenheit, mein Fahrrad mitzunehmen	3,16 (0,75)	2,57 (1,51)	3,14 (0,89)	3,14 (0,69)	3,20 (0,78)
*11*	In meiner Wohnumgebung gibt es viele sichere Fahrrad-Abstellanlagen	3,0 (1,41)	3,14 (1,46)	2,85 (1,34)	2,85 (1,34)	2,96 (1.16)
*12*	In meiner Wohnumgebung fühle ich mich beim Radfahren vor Kriminalität sicher^1^	2,42 (0,78)	2,0 (1,15)	2,57 (1,27)	2,14 (1,06)	2,28* (0,91)
*13*	In meiner Wohnumgebung fühle ich mich aufgrund der Verkehrssituation beim Radfahren sicher	3,42 (1,13)	3,28 (1,11)	3,0 (1)	2,71 (1,11)	3,10 (0,87)
*14*	Meine Wohnumgebung ist eine schöne Umgebung, um Fahrrad zu fahren	2,83 (1,47)	2,42 (1,51)	3,28 (0,95)	2,0 (0,81)	2,75 (0,88)
*15*	In meiner Wohnumgebung gibt es viele Anstiege oder Hügel^1^	2,42 (1,27)	3,14 (1,57)	3,42 (0,53)	2,57 (0,78)	2,89 (0,51)
*16*	In meiner Wohnumgebung sind die Ampeln so geschaltet, dass ich als Radfahrer:in zügig vorankomme	3,85 (0,37)	3,14 (1,57)	3,28 (0,75)	2,71 (0,75)	3,25 (0,52)
*17*	In meiner Wohnumgebung fahren viele Menschen mit dem Fahrrad	3,28 (0,95)	3,0 (1,29)	3,28 (0,95)	3,0 (0,81)	3,14 (0,93)
*18*	In meiner Wohnumgebung ist die Radweginfrastruktur in den letzten fünf Jahren deutlich verbessert worden^1^	2,50 (1,37)	2,0 (1,41)	2,57 (1,39)	2,14 (1,34)	2,41* (1,31)
*19*	Ich bewerte meine Wohnumgebung insgesamt als fahrradfreundlich	3,57 (0,53)	3,71 (0,48)	3,71 (0,48)	3,14 (0,89)	3,53 (0,50)

*M_gesamt_ < 2,70

^1^Item entfernt

### Kognitives Testen

Im Folgenden sind die Ergebnisse der kognitiven Interviews (*n* = 18) dargestellt. Die durchschnittliche Dauer der Interviews betrug 49:55 min.

#### Merkmale der Stichprobe.

Insgesamt nahmen 18 Personen an den kognitiven Interviews teil. Tab. 5 zeigt die Verteilung der Stichprobe nach Alter, Migrationshintergrund, Bildungsabschluss, Bundesland sowie Wohnlage. Beim Migrationshintergrund wurde zusätzlich angegeben, ob nur ein Elternteil oder beide Elternteile der befragten Person in einem anderen Land geboren sind.

**Tab. 5:** Verteilung der Stichprobe der kognitiven Interviews (*N* = 18)

Merkmal	Ausprägung	Gesamt	Weiblich	Männlich
Alter	18–29 Jahre (junge Erwachsene)	6	3	3
	45–64 Jahre (Erwachsene im späten mittleren Alter)	4	2	2
	30–44 Jahre (Erwachsene im frühen mittleren Alter)	7	3	4
	65–79 Jahre (ältere Erwachsene)	1	1	0
	> 80 Jahre (Hochbetagte)	0	0	0
Migrationshintergrund	Migrant:in (ein Elternteil)	2	0	2
	Migrant:in (beide Elternteile)	1	1	0
	Ohne Migrationshintergrund	15	8	7
Bildungsabschluss	Kein Abschluss	0	0	0
	Qualifizierter Mittelschulabschluss	1	0	1
	Mittlere Reife	2	2	0
	Allgemeine Hochschulreife	2	0	2
	Staatsexamen	2	0	2
	Diplom	1	1	0
	Bachelor	2	1	1
	Master	4	3	1
	Promotion	4	2	2
Bundesland	Bayern	8	4	4
	Niedersachsen	1	0	1
	Berlin	9	5	4
Wohnlage	Innenstadtlage in einer Großstadt (> 100.000 Einwohner)	6	4	2
	Stadtrandlage in einer Großstadt (> 100.000 Einwohner)	8	5	3
	Vorort von einer Großstadt (> 100.000 Einwohner)	1	0	1
	Stadt mittlerer Größe (20.000 bis 100.000 Einwohner)	0	0	0
	Kleinstadt (5000 bis 20.000 Einwohner)	1	0	1
	Ländlich/Dorf (< 5000 Einwohner)	2	0	2

#### Ergebnisse des kognitiven Testens

Für die Beantwortung des überarbeiteten Walkability-Instruments benötigten die interviewten Personen im Durchschnitt 2:10 min und für die Beantwortung des überarbeiteten Bikeability-Instruments 2:39 min.

Insgesamt zeigen die Ergebnisse des kognitiven Testens, dass beide Instrumente von der Mehrheit der Befragten als leicht verständlich empfunden wurden. Beispielsweise antworteten 16 von 18 befragten Personen auf die Nachfrage, ob es leicht oder schwer gewesen sei, die Frage 3 des Walkability-Instruments („In meiner Wohnumgebung wird den Fußgänger:innen genug Raum gegeben und die Gehwege sind ausreichend breit.“) zu beantworten, dass es ihnen leichtgefallen sei, diese Frage zu beantworten. Gleiches zeigt sich auch bei den Fragen 13 und 14 des Bikeability-Instruments. Hier bewerteten 15 Personen (Frage 13) bzw. 14 Personen (Frage 14) die Fragen mit „leicht zu beantworten“.

Fünf Befragte berichteten, dass ihnen die Beantwortung des Walkability-Instruments leichter gefallen sei als die Beantwortung des Bikeability-Instruments, bei 7 Personen war es umgekehrt und die übrigen 7 Befragten stellten keine Unterschiede fest. 15 von den insgesamt 18 befragten Personen fanden die einleitende Beschreibung leicht verständlich.

Es zeigt sich, dass 8 (Walkability) bzw. 9 (Bikeability) von 18 interviewten Personen aufgrund der Subjektivität einzelner Begrifflichkeiten Schwierigkeiten mit dem Verständnis (Bereich „Comprehension“) hatten. Kritisiert wurde, dass Begriffe wie „viel“, „schön“, „Gehweginfrastruktur“ oder „genug Raum“ unterschiedlich interpretiert und bewertet werden könnten, wenn eine genaue Definition fehlt. Insbesondere ging es hierbei um die Fragen 2, 3, 6, 9, 11 und 12 des Walkability-Instruments und die Fragen 2, 3, 4, 9, 10, 11 und 13 des Bikeability-Instruments. Die übrigen 10 bzw. 9 Personen hatten keine Verständnisprobleme.

Im Bereich Urteil (Judgment) zeigt sich insgesamt, dass 10 von 18 befragten Personen keine Schwierigkeiten äußerten und somit insgesamt eine hohe Gewissheit für korrekt gegebene Antworten bei beiden Instrumenten vorliegt. Einige Befragte berichteten Schwierigkeiten bei den Fragen 3, 6 und 11 des Walkability-Instruments und bei den Fragen 11, 13 und 14 des Bikeability-Instruments. Bei Frage 6 des Walkability-Instruments vermuteten 8 Befragte, dass Personen, die in einer Wohnumgebung leben, die sie nicht selbst gewählt haben und in der sie sich unsicher fühlen, diese Frage möglicherweise nicht ehrlich beantworten würden und es so zu Unwohlsein aufseiten der Befragten bei der Beantwortung dieser Frage kommen könnte. Weitere 8 Personen äußerten bei Frage 11, dass sie bei dieser Frage länger überlegen mussten als bei anderen Fragen.

Hinsichtlich der Aspekte Abruf bzw. Erinnerung (Retrieval) berichteten 15 von 18 befragten Personen, dass sie keinerlei Probleme beim Abrufen der Informationen zur Beantwortung der Fragen hatten. Drei Befragte berichteten von Schwierigkeiten bei den Fragen 6 und 9 des Walkability-Instruments, während beim Bikeability-Instrument 2 Personen die Frage 7 als schwer verständlich einstuften. Wie bereits im Bereich Verständnis bezogen sich diese Probleme lediglich auf die Subjektivität oder fehlende Definitionen zu einzelnen Begrifflichkeiten, wie beispielsweise „Kriminalität“ oder „Verkehrssituation“.

Im Bereich Antwortverhalten (Response) zeigen sich keine Schwierigkeiten bei den Befragten.

Abschließend wurden die interviewten Personen gefragt, ob sie sich an eine Frage erinnerten, deren Beantwortung sie besonders schwierig fanden. Sechs Personen gaben an, dass sie keine der in den beiden Befragungsinstrumenten gestellten Fragen schwierig fanden. Zwei Personen berichteten, dass sie Schwierigkeiten hatten, alle Fragen, in denen Wörter wie „viel“ und „schön“ vorkamen, zu beantworten. Zwei weitere Personen gaben an, dass sie Frage 10 des Bikeability-Instruments zur Verkehrssicherheit schwierig fanden. Die restlichen 8 Befragten äußerten, dass sie keine der gestellten Fragen schwierig fanden.

Auf Grundlage der Ergebnisse des kognitiven Testens wurde in beiden Befragungsinstrumenten eine fünfte Antwortkategorie „weiß nicht/keine Angabe“ ergänzt. Zudem wurde für das Item zur Barrierefreiheit des Walkability-Instruments auf Vorschlag einer interviewten Person der Begriff „barrierefrei“ durch „barrierearm“ ersetzt. Die finalen Instrumente sind Onlinematerial 3 und 4 zu entnehmen.

## Diskussion

Die Länder können mit den entwickelten Befragungsinstrumenten bevölkerungsbezogene Daten für die beiden Präventionsindikatoren Walkability und Bikeability erheben. Die Indikatoren können im Rahmen der Nationalen Präventionsstrategie eine wichtige Evidenzgrundlage für den alle 4 Jahre erscheinenden Nationalen Präventionsbericht darstellen. Ferner können die Länder die Indikatoren für die eigene Präventionsberichterstattung zur Evaluation der Landesrahmenvereinbarungen einsetzen.

Die Instrumente wurden in Anlehnung an die bereits existierenden Instrumente NEWS‑G, ALPHA und PANES als Kurzbefragungsinstrumente entwickelt und an den deutschen Kontext adaptiert. Eine Befragung von Expertinnen und Experten und eine Studie zur kognitiven Testung dienten der Überprüfung und Adaption der Instrumente. Die Befragung diente der Entwicklung, Überprüfung, Ergänzung, Priorisierung und Kürzung der Item-Listen. Die Instrumente zeigen im kognitiven Testen insgesamt gute Ergebnisse hinsichtlich Verständlichkeit, Einfachheit, Gedankenprozess beim Ausfüllen, Geeignetheit der Antwortkategorien und Sicherheit mit den gegebenen Antworten, sodass sie in einem nächsten Schritt in einer Pilotdatenerhebung eingesetzt werden können.

Die Ergebnisse der kognitiven Interviews zeigen, dass die Instrumente mehrheitlich als verständlich und einfach auszufüllen bewertet wurden und dass die Teilnehmenden bei der Beantwortung der Fragen wie intendiert vorgegangen sind. 15 von 18 Befragten fanden die Anweisungen zum Ausfüllen der Instrumente verständlich. Die meisten berichteten Schwierigkeiten und kritischen Rückmeldungen der Befragten betrafen die Subjektivität und die fehlenden Definitionen einzelner Begrifflichkeiten. Einige Teilnehmende äußerten daher den Wunsch nach einem einheitlichen Begriffsverständnis, nach konkreten Beispielen oder vorgegebenen Definitionen innerhalb der Befragungsinstrumente. Umfassende Definitionen und Beispiele in die Fragentexte zu integrieren, hat den Nachteil, dass die Fragen dadurch zu umfangreich und unverständlich werden. Begriffe wie „gut ausgebaut“ oder „viele“ sind per se relativ und kontextabhängig, da sie stark von individuellen Faktoren und Erfahrungen, Erwartungen und dem sozialen Umfeld der Befragten beeinflusst und daher in subjektiven Befragungen derart intendiert sind [10, 11, 13]. Dies kann einerseits die Reliabilität der Instrumente reduzieren, andererseits aber auch wertvolle Einblicke in die subjektive Wahrnehmung der Befragten ermöglichen. Die Subjektivität der Items der Instrumente steht nicht im Widerspruch zu dem angestrebten Ziel der Befragung, die wahrgenommene Walkability bzw. Bikeability der Wohnumgebung zu erfassen.

Neben der Kritik hinsichtlich der Subjektivität äußerten einige der Befragten den Wunsch nach einer fünften, neutralen Antwortkategorie (z. B. „neutral“) oder einer Ausweichoption („weiß nicht/keine Angabe“). Eine fünfte Antwortkategorie „weiß nicht/keine Angabe“ wurde entsprechend in beiden Instrumenten eingefügt, um den Teilnehmenden die Möglichkeit zu geben, Fragen unbeantwortet zu lassen, falls für sie keine andere Antwortoption eindeutig passend ist oder die Frage auf ihre Wohnumgebung nicht zutrifft. Diese fünfte Antwortoption verfolgt das Ziel, das Risiko von Verzerrungen aufgrund von Unentschlossenheit zu reduzieren. Gleichzeitig wurde bewusst auf eine mittlere, neutrale Antwortoption verzichtet, um einer „Tendenz zur Mitte“ entgegenzuwirken, die als Verzerrungsquelle bei Befragungen gilt [23]. Abgesehen von dieser Ergänzung wurden keine weiteren Änderungen an den Instrumenten vorgenommen.

Zusammenfassend ist festzustellen, dass sich aufgrund der intendierten Erhebungsziele der Instrumente aus der geäußerten Kritik an den Itemformulierungen und dem Wunsch nach einer mittleren Antwortoption keine weiteren Adaptionen ableiten lassen. In zukünftigen Studien könnten je nach Fragestellung ergänzende objektive Messungen einbezogen werden, um die subjektiven Einschätzungen zu kontextualisieren. Insgesamt haben die Ergebnisse der Befragung von Expertinnen und Experten und des kognitiven Testens sowie die vorgenommenen Anpassungen dazu beigetragen, neue Kurzinstrumente für die Erfassung der subjektiven Wahrnehmung von Walkability und Bikeability bereitzustellen und gleichzeitig die methodischen Herausforderungen hierbei transparent zu machen.

### Limitationen

Die vorliegende Studie weist einige Limitationen auf. Zum einen ist die Stichprobenanzahl der Expertenbefragung gering. Aufgrund der Komplexität sowie des Umfangs der Befragung war es nicht gelungen, mehr Expertinnen und Experten für eine Teilnahme zu gewinnen. Zum anderen weist die Stichprobe des kognitiven Testens nur eine geringe Heterogenität auf, da lediglich Probandinnen und Probanden aus 2 Städten (Erlangen und Berlin) gewonnen werden konnten. Zudem haben nur 3 von insgesamt 18 Befragten einen Migrationshintergrund und nur 3 Personen einen niedrigeren Schulabschluss als das Abitur. Schließlich kann die Methode des kognitiven Testens hinsichtlich ihrer Ergebnisse Limitationen aufweisen. Beispielsweise können Schwächen des Instruments für die untersuchten Personen bei der kognitiven Testung unentdeckt bleiben oder die Relevanz der beim kognitiven Testen festgestellten Schwächen überschätzt werden [24]. Es ist zu beachten, dass die hier dargestellten Instrumente zum aktuellen Zeitpunkt noch nicht in Bezug auf alle wissenschaftlichen Standards geprüft wurden. Die Überprüfung beispielsweise der Test-Retest-Reliabilität bzw. der Kriteriums- oder Konstruktvalidität steht noch aus. Dennoch ist hervorzuheben, dass die beiden hier vorgestellten Instrumente in Anlehnung an die 3 bereits validierten Skalen NEWS‑G, ALPHA sowie PANES entwickelt und lediglich an den deutschen Kontext angepasst wurden [10, 11, 13].

## Fazit

Durch die Expertenbefragung konnten sowohl das Walkability- als auch das Bikeability-Instrument gekürzt werden. Die Ergebnisse des kognitiven Testens zeigen, dass beide Instrumente insgesamt als gut verständlich und einfach anzuwenden wahrgenommen wurden. Es zeigte sich, dass einzelne Begrifflichkeiten, wie beispielsweise „viel“, „schön“ oder „gut ausgebaut“, in hohem Maß subjektiven Einschätzungen unterliegen, was allerdings intendiert wird und mit den zu erfassenden Konstrukten in Einklang steht. Zur Verbesserung der Antwortqualität wurde auf Grundlage der Antworten in den kognitiven Interviews eine zusätzliche Antwortoption eingefügt („weiß nicht/keine Angabe“), die das Risiko von Verzerrungen minimieren soll.

Insgesamt bieten die beiden im Rahmen des vorliegenden Projekts entwickelten Instrumente einen empirisch fundierten Ansatz zur Erfassung der subjektiven Wahrnehmung der beiden Präventionsindikatoren Walkability und Bikeability im deutschsprachigen Raum, welche sich aufgrund ihrer Verständlichkeit und Kürze insbesondere für die Anwendung in bevölkerungsbezogenen Studien sowie für die Präventionsberichterstattung der Länder eignen. An dieser Stelle ist zu berücksichtigen, dass die hier dargestellten Instrumente zum jetzigen Zeitpunkt noch nicht in Bezug auf alle wissenschaftlichen Standards geprüft wurden. Es wird angestrebt, im Anschluss an die Expertenbefragung sowie das kognitive Testen die finalen Instrumente in einer bevölkerungsbezogenen Befragung in Berlin zu pilotieren und schließlich in einer bundesweiten Befragung einzusetzen, in deren Rahmen auch die Test-Retest-Reliabilität überprüft werden könnte.

## Supplementary Information

Onlinematerial 1: Interviewleitfaden kognitives Testen

Onlinematerial 2: Survey Answers

Onlinematerial 3: Walkability

Onlinematerial 4: Bikeability

## Data Availability

Die während der vorliegenden Studie erhobenen und/oder analysierten Datensätze sind auf begründete Nachfrage bei der Korrespondenzperson erhältlich.

## References

[1] de Walque D (2020) The use of financial incentives to prevent unhealthy behaviors: A review. Soc Sci Med 261:113236. 10.1016/j.socscimed.2020.113236 32781370 10.1016/j.socscimed.2020.113236

[2] Candari CJ, Cylus J, Nolte E. Assessing the economic costs of unhealthy diets and low physical activity: An evidence review and proposed framework [Internet]. Copenhagen (Denmark): European Observatory on Health Systems and Policies; 2017. PMID: 28787114. Abgerufen unter: https://www.ncbi.nlm.nih.gov/books/NBK447219 28787114

[3] Zhong J, Liu W, Niu B, Lin X, Deng Y (2022) Role of Built Environments on Physical Activity and Health Promotion: A Review and Policy Insights. Front Public Health 10:950348. 10.3389/fpubh.2022.950348 35910910 10.3389/fpubh.2022.950348 PMC9326484

[4] Nationale Präventionskonferenz (NPK) Zweiter Präventionsbericht nach § 20d Abs. 4 SGB V. Berlin: NPK; 2023. Abgerufen unter: https://www.npk-info.de/fileadmin/user_upload/ueber_die_npk/downloads/2_praeventionsbericht/zweiter_npk_praeventionsbericht_barrierefrei.pdf

[5] Bucksch J, Schneider S (2014) Walkability. Das Handbuch zur Bewegungsförderung in der Kommune. Huber, Bern

[6] Grasser G, Van Dyck D, Titze S, Stronegger W (2013) Objectively measured walkability and active transport and weight-related outcomes in adults: a systematic review. Int J Public Health 58:615–625. 10.1007/s00038-012-0435-0 23224518 10.1007/s00038-012-0435-0

[7] Shareck M, Fuller D, Sersli S et al (2023) Measuring walkability and bikeability for health equity and intervention research: a scoping review. Cities Health 7:1108–1117. 10.1080/23748834.2023.2260133

[8] Kellstedt DK, Spengler JO, Foster M, Lee C, Maddock JE (2021) A Scoping Review of Bikeability Assessment Methods. J Community Health 46:211–224. 10.1007/s10900-020-00846-4 32419079 10.1007/s10900-020-00846-4

[9] Iroz-Elardo N, Adkins A, Ingram M (2021) Measuring perceptions of social environments for walking: A scoping review of walkability surveys. Health Place 67:102468. 10.1016/j.healthplace.2020.102468 33285411 10.1016/j.healthplace.2020.102468

[10] Bödeker M, Bucksch J, Fuhrmann H (2012) Bewegungsfreundlichkeit von Wohnumgebungen messen. Präv Gesundheitsf 7:220–226. 10.1007/s11553-012-0344-3

[11] Bucksch J, Spittaels H (2011) Reliability and validity findings of the ALPHA environmental questionnaire in Germany. J Public Health 19:417–423. 10.1007/s10389-011-0416-4

[12] Tristram C, Till M, Finger JD, John S, Manz K, Reimers AK (2025) Befragungsinstrumente zur subjektiven Erfassung von Walkability und Bikeability – ein Rapid Review. Präv Gesundheitsf. 10.1007/s11553-025-01268-710.1007/s11553-025-01268-7

[13] Sallis JF, Kerr J, Carlson JA et al (2010) Evaluating a brief self-report measure of neighborhood environments for physical activity research and surveillance: Physical Activity Neighborhood Environment Scale (PANES). J Phys Act Health 7:533–540. 10.1123/jpah.7.4.533 20683096 10.1123/jpah.7.4.533

[14] Moosburger H, Kelava A (2012) Testtheorie und Fragebogenkonstruktion. Springer, Heidelberg

[15] Bogner A, Littig B, Menz W (2002) Das Experteninterview. Theorie, Methode, Anwendung. VS Verlag für Sozialwissenschaften. Wiesbaden

[16] Lenzner T, Neuert C, Otto W. Kognitives Pretesting. Mannheim: GESIS – Leibniz-Institut für Sozialwissenschaften; 2015. GESIS Survey Guidelines. 10.15465/gesis-sg_010. Abgerufen unter: https://www.gesis.org/fileadmin/admin/Dateikatalog/pdf/guidelines/kognitives_pretesting_lenzner_neuert_otto_2015.pdf

[17] Pohontsch N, Meyer T (2015) Cognitive interviewing - a tool to develop and validate questionnaires. Rehabil 54:53–59. 10.1186/s13690-025-01661-w 25675322 10.1055/s-0034-1394443

[18] Schaefer I, Kolip P. Leitfaden Goal Attainment Scaling (GAS) – Zielerreichungsskalen. Düsseldorf: Landeszentrale für Gesundheitsförderung Nordrhein-Westfalen; 2011. Abgerufen unter: https://www.lzg.nrw.de/_media/pdf/service/Veranst/110705_Workshop_Zielerreichungsskalen/leitfaden_gas_endversion.pdf

[19] Tourangeau R, Rips LJ, Rasinski K (2000) The psychology of survey response. Cambridge University Press, Cambridge

[20] DeMaio TJ, Landreth A. Do different cognitive interview techniques produce different results? In: Presser S, Rothgeb JM, Couper MP, Lessler JT, Martin E, Martin J, Singer E, editors. Methods for Testing and Evaluating Survey Questionnaires. Hoboken (NJ): John Wiley & Sons; 2004. S. 89-108. 10.1002/0471654728.ch5

[21] Rodrigue L, Daley J, Ravensbergen L et al (2022) Factors influencing subjective walkability: Results from built environment audit data. JTLU 15:709–727. 10.5198/jtlu.2022.2234

[22] Cairns S, Behrendt F, Raffo D, Beaumont C, Kiefer C (2017) Electrically-assisted bikes: Potential impacts on travel behaviour. Transportation Res Part A: Policy Pract 103:327–342. 10.1016/j.tra.2017.03.007

[23] Bogner K, Landrock U. Antworttendenzen in standardisierten Umfragen. Mannheim: GESIS – Leibniz-Institut für Sozialwissenschaften; 2015. GESIS Survey Guidelines. 10.15465/gesis-sg_016. Abgerufen unter: https://www.gesis.org/fileadmin/admin/Dateikatalog/pdf/guidelines/antworttendenzen_bogner_landrock_2015.pdf

[24] Beatty PC, Willis GB (2007) Research Synthesis: The Practice of Cognitive Interviewing. PUBOPQ 71:287–311. 10.1093/poq/nfm006

