# Supplementary material for: Development of survey instruments for assessing walkability and bikeability for the prevention indicator system of the German federal states
Source: Bundesgesundheitsblatt Gesundheitsforschung Gesundheitsschutz. 2025 Dec 2;69(1):51–60. [Article in German] doi: 10.1007/s00103-025-04163-w (PMC12764655; doi:10.1007/s00103-025-04163-w)
Supplement: Supplementary file 1 — Onlinematerial 1: Interviewleitfaden kognitives Testen [file 103_2025_4163_MOESM1_ESM.pdf]

<u>Onlinematerial 1: Interviewleitfaden Kognitives Testen</u>

Ziele des Interviews:

- Verständlichkeit der Fragen der beiden Fragebögen zu Walkability und Bikeability testen.
- Einschätzung von zeitlichem Aufwand für die Beantwortung der beiden Fragebögen.

Untersuchungsfragen:

- Überschneiden sich die beiden Skalen Walkability und Bikeability inhaltlich?
- Gibt es innerhalb der Fragenkomplexe inhaltliche Überschneidungen zwischen den Fragen innerhalb eines Fragebogens?
- Verstehen die Teilnehmenden die Frageformulierungen und einzelne Begrifflichkeiten?
- Gab es eine oder mehrere Fragen, bei denen sich die Teilnehmenden unwohl gefühlt haben?
- Wie haben die Teilnehmenden die Antwortkategorien genutzt? Fanden sie es einfach oder schwer, die Abstufungen der Likert-Skala zu unterscheiden?
- Wie legen die Teilnehmenden ihre Antworten fest bzw. wie lange brauchen sie für die Beantwortung einzelner Fragen und den beiden Gesamtskalen?

[START]

Liebe/r Teilnehmer/in,

vielen Dank, dass Sie sich die Zeit nehmen um an unserer Studie teilzunehmen. Ich werde nun die Aufnahme starten. Es wird nur der Ton aufgezeichnet, nicht das Bild.

[TONAUFNAHME STARTEN]

Sind Sie einverstanden mit der Teilnahme an diesem Interview und der Tonaufnahme?

[WENN JA, WEITER]

*Ich möchte Sie an dieser Stelle noch einmal darauf hinweisen, dass Sie das Interview jederzeit abbrechen dürfen. Dadurch entstehen für Sie keinerlei Nachteile. Ebenso haben Sie das Recht, einzelne Fragen des Interviews nicht zu beantworten. Dann gehen wir einfach zu der nächsten Frage über. Das Interview gliedert sich in zwei Abschnitte. In dem ersten Abschnitt beschäftigen wir uns mit dem Konzept der Walkability. Walkability bedeutet so viel wie Fußgänger*innenfreundlichkeit, also wie attraktiv eine Umgebung zum zu Fuß gehen ist. Im zweiten Abschnitt beschäftigen wir uns mit Bikeability, also wie freundlich eine Umgebung zum Radfahren ist. Um zu erfahren, wie fußgänger*innenfreundlich bzw. radfahrfreundlich Menschen ihre Wohnumgebung wahrnehmen, haben wir zwei Fragebögen entwickelt, mit denen wir die Wahrnehmung von Walkability und Bikeability abfragen können. Das Ziel dieses Interviews ist es herauszufinden, wie verständlich diese beiden Fragebögen sind. Zuerst würden wir Sie bitten, den Walkability-Fragebogen einmal selbst auszufüllen. Wenn Sie fertig sind, stelle ich Ihnen ein paar Fragen zu diesem Fragebogen. Danach würde ich Sie bitten, den Bikability-Fragebogen auszufüllen. Auch hier werde ich Ihnen im Anschluss Fragen zu diesem Fragebogen stellen. Sie haben ausreichend Zeit, die beiden Fragebögen zu Walkability und Bikeability zu beantworten. Gibt es von Ihrer Seite noch Fragen zum Vorgehen oder unserem Interview allgemein?*

[WENN NEIN, WEITER]

Bevor wir mit der Beantwortung der beiden Fragebögen und anschließenden Nachfragen starten, benötige ich ein paar Angaben zu Ihrer Person.

1) Soziodemografische Angaben

1.1) Wie alt sind Sie?
1.2) Was ist Ihr Geschlecht?
1.3) Was ist ihr höchster Bildungsabschluss? Zum Beispiel Berufsausbildung, Bachelor, Master etc.
1.4) Was beschreibt Ihre Wohnlage am besten?

- Innenstadtlage in einer Großstadt
- Stadtrandlage in einer Großstadt
- Vorort von einer Großstadt
- Stadt mittlerer Größe
- Kleinstadt
- Ländlich / Dorf

[WALKABILITY]

Vielen Dank, dann starten wir mit dem Walkability-Fragebogen. Bitte füllen Sie diesen einmal aus. Bitte nehmen Sie sich hierfür ausreichend Zeit.

[ZEIT STOPPEN ZUM AUSFÜLLEN DES FRAGEBOGENS – START]

[AUSFÜLLEN LASSEN]

<u>Zu Fuß gehen in Ihrer Wohnumgebung</u>

Mit den nachfolgenden Fragen möchten wir erfahren, wie fußgänger*innenfreundlich Ihre unmittelbare Wohnumgebung ist.

Denken Sie nun an Ihre unmittelbare Wohnumgebung um Ihren Wohnsitz, die zu Fuß in 10 – 15 Minuten zu erreichen ist. Bitte markieren Sie zu den unten aufgeführten Aussagen jeweils die aus Ihrer Sicht zutreffendste Antwort.

	Stimme überhaupt nicht zu	Stimme eher nicht zu	Stimme eher zu	Stimme vollständig zu
1. In meiner Wohnumgebung gibt es viele Einrichtungen des täglichen Bedarfs wie Geschäfte, Restaurants, Apotheken, Freizeiteinrichtungen und Schulen, die innerhalb von 10 - 15 min zu Fuß zu erreichen sind.	☐	☐	☐	☐
2. In meiner Wohnumgebung gibt es eine gut ausgebaute Gehweginfrastruktur.	☐	☐	☐	☐
3. In meiner Wohnumgebung wird den Fußgänger*innen genug Raum gegeben und die Gehwege sind ausreichend breit.	☐	☐	☐	☐
4. In meiner Wohnumgebung sind die Gehwege in einem guten Zustand.	☐	☐	☐	☐
5. In meiner Wohnumgebung kann ich Haltestellen des öffentlichen Nahverkehrs (Bus/Bahn) gut zu Fuß erreichen.	☐	☐	☐	☐
6. In meiner Wohnumgebung fühle ich mich beim zu Fuß gehen vor Kriminalität sicher.	☐	☐	☐	☐
7. In meiner Wohnumgebung gibt es viele verkehrsberuhigte Abschnitte wie 30er Zonen, Spielstraßen oder Fußgängerzonen.	☐	☐	☐	☐

8. In meiner Wohnumgebung gibt es viele Zebrastreifen, Fußgängerampeln, Brücken oder Unterführungen.	☐	☐	☐	☐
9. In meiner Wohnumgebung fühle ich mich aufgrund der Verkehrssituation beim zu Fuß gehen sicher.	☐	☐	☐	☐
10. Meine Wohnumgebung ist eine schöne Umgebung um zu Fuß zu gehen.	☐	☐	☐	☐
11. In meiner Wohnumgebung sind die Gehwege barrierefrei, beispielsweise gibt es abgesenkte Bordsteine, Bodenleitsysteme oder Ampeln mit Tonsignal.	☐	☐	☐	☐
12. In meiner Wohnumgebung gehen viele Menschen zu Fuß.	☐	☐	☐	☐
13. Ich bewerte meine Wohnumgebung insgesamt als fußgänger*innenfreundlich.	☐	☐	☐	☐

[ZEIT STOPPEN – ENDE]

Notes für Beobachtungen:

Wieviel Zeit wurde zum Ausfüllen des Fragebogens benötigt?

[NACHFRAGEN]

Sie sind nun fertig mit dem Ausfüllen des Walkability-Fragebogens. Nun werde ich Ihnen spezifisch Nachfragen zu einzelnen Fragen der Walkability-Skala stellen. Bitte beantworten Sie diese nach bestem Gewissen.

2) Spezifische Fragen zum Walkability-Fragebogen

<u>Q2: In meiner Wohnumgebung gibt es eine gut ausgebaute Gehweginfrastruktur.</u>

2.1) Was bedeutet der Begriff „Gehweginfrastruktur“ für Sie? Fallen Ihnen dafür Synonyme ein?

2.2) Was bedeutet für Sie „gut ausgebaut“?

2.3) Woran haben Sie gedacht, als Sie diese Frage beantwortet haben? Nennen Sie gern Beispiele.

<u>Q3: In meiner Wohnumgebung wird den Fußgänger*innen genug Raum gegeben und die Gehwege sind ausreichend breit.</u>

2.4) Was bedeutet für Sie „genug Raum“?

2.5) Wie definieren Sie „ausreichend breit“?

2.6) Fiel es Ihnen leicht oder schwer diese Frage zu beantworten?

Q4: In meiner Wohnumgebung sind die Gehwege in einem guten Zustand.

2.7) An was für Gehwege denken Sie, wenn sich diese „in einem guten Zustand“ befinden? Beschreiben Sie gern.

Q6: In meiner Wohnumgebung fühle ich mich beim zu Fuß gehen vor Kriminalität sicher.

2.8) Beschreiben Sie, wie Ihre Wohnumgebung aussehen muss, damit Sie sich dort vor Kriminalität sicher fühlen.

2.9) Finden Sie, dass diese Frage zu persönlich ist oder denken Sie, dass es in Ordnung ist, eine solche Frage in einer Umfrage gestellt zu bekommen?

2.10) Denken Sie, dass manche Menschen möglicherweise keine ehrliche Antwort auf diese Frage geben würden?

Q9: In meiner Wohnumgebung fühle ich mich aufgrund der Verkehrssituation beim zu Fuß gehen sicher.

2.11) Beschreiben Sie, wie Ihre Wohnumgebung aussehen muss, damit Sie sich dort sicher im Verkehr bewegen können.

2.12) Können Sie mir in Ihren eigenen Worten sagen, worum es in dieser Frage geht?

Q10: Meine Wohnumgebung ist eine schöne Umgebung um zu Fuß zu gehen.

2.13) Was bedeutet für Sie eine „schöne Umgebung“ zum zu Fuß gehen?

2.14) Ihre Antwort bei dieser Frage war X. Wieso haben Sie sich für diese Antwort entschieden, und nicht für einen höheren oder niedrigeren Wert?

2.15) War es einfach oder schwer sich hier für eine Antwortmöglichkeit zu entscheiden?

Q11: In meiner Wohnumgebung sind die Gehwege barrierefrei, beispielsweise gibt es abgesenkte Bordsteine, Bodenleitsysteme oder Ampeln mit Tonsignal.

2.16) Wie leicht oder schwer ist es Ihnen gefallen diese Frage zu beantworten?

2.17) Wie definieren Sie den Begriff „barrierefrei“? Fallen Ihnen dafür Synonyme ein?

Q12: In meiner Wohnumgebung gehen viele Menschen zu Fuß.

2.18) Wie definieren Sie hier „viel“?

2.19) Wie sind Sie bei der Beantwortung dieser Frage vorgegangen, um sich für eine Antwort zu entscheiden? Beschreiben Sie gern.

[BIKEABILITY]

Den Fragebogen zu Walkability haben wir nun abgeschlossen. Daher geht es jetzt weiter mit dem Fragebogen zu Bikeability. Wir starten nun mit der Beantwortung des Bikeability-Fragebogens. Bitte nehmen Sie sich auch hier ausreichend Zeit, um den Fragebogen auszufüllen.

[ZEIT STOPPEN ZUM AUSFÜLLEN DES FRAGEBOGENS – START]

[AUSFÜLLEN LASSEN]

Fahrrad fahren in Ihrer Wohnumgebung

Mit den nachfolgenden Fragen möchten wir erfahren, wie radfahrfreundlich Ihre unmittelbare Wohnumgebung ist.

Denken Sie nun an Ihre unmittelbare Wohnumgebung um Ihren Wohnsitz, die mit dem Fahrrad in 10 – 15 Minuten zu erreichen ist. Bitte markieren Sie zu den unten aufgeführten Aussagen jeweils die aus Ihrer Sicht zutreffendste Antwort.

	Stimme überhaupt nicht zu	Stimme eher nicht zu	Stimme eher zu	Stimme vollständig zu
1. In meiner Wohnumgebung gibt es viele Einrichtungen des täglichen Bedarfs wie Geschäfte, Restaurants, Apotheken, Freizeiteinrichtungen und Schulen, die innerhalb von 10 - 15 min mit dem Fahrrad zu erreichen sind.	☐	☐	☐	☐
2. In meiner Wohnumgebung gibt es viele ausgewiesene Radstreifen und Radwege.	☐	☐	☐	☐
3. In meiner Wohnumgebung gibt es viele ausgewiesene Radschnellwege und Fahrradstraßen.	☐	☐	☐	☐
4. In meiner Wohnumgebung wird den Radfahrer*innen genug Raum gegeben und die Radwege sind ausreichend breit.	☐	☐	☐	☐
5. In meiner Wohnumgebung sind die Radwege in einem guten Zustand.	☐	☐	☐	☐
6. In meiner Wohnumgebung gibt es Radwege, die vom Verkehr getrennt sind.	☐	☐	☐	☐
7. In meiner Wohnumgebung kann ich Haltestellen des öffentlichen Nahverkehrs (Bus/Bahn) gut mit dem Fahrrad erreichen.	☐	☐	☐	☐
8. Der öffentliche Nahverkehr (Bus/Bahn) in meiner Wohnumgebung bietet mir Gelegenheit, mein Fahrrad mitzunehmen.	☐	☐	☐	☐
9. In meiner Wohnumgebung gibt es viele sichere Fahrrad-Abstellanlagen.	☐	☐	☐	☐
10. In meiner Wohnumgebung fühle ich mich aufgrund der Verkehrssituation beim Radfahren sicher.	☐	☐	☐	☐
11. Meine Wohnumgebung ist eine schöne Umgebung um Fahrrad zu fahren.	☐	☐	☐	☐
12. In meiner Wohnumgebung sind die Ampeln so geschaltet, dass ich als Radfahrer*in zügig vorankomme.	☐	☐	☐	☐
13. In meiner Wohnumgebung fahren viele Menschen mit dem Fahrrad.	☐	☐	☐	☐

14. Ich bewerte meine Wohnumgebung insgesamt als radfahrfreundlich.	☐	☐	☐	☐

[ZEIT STOPPEN – ENDE]

Notes für Beobachtungen:

Wieviel Zeit wurde zum Ausfüllen des Fragebogens benötigt?

[NACHFRAGEN]

Sie sind nun fertig mit dem Ausfüllen des Bikeability-Fragebogens. Wie bereits zuvor beim Walkability-Fragebogen werde ich Ihnen nun Nachfragen zu einzelnen Fragen der Bikeability-Skala stellen. Bitte beantworten Sie auch hier die nachfolgenden Fragen nach bestem Wissen.

3) Spezifische Fragen zum Bikeability-Fragebogen

<u>Q2: In meiner Wohnumgebung gibt es viele ausgewiesene Radstreifen und Radwege.</u>

3.1) Wie definieren Sie hier „viel“?

3.2) Beschreiben Sie, was für Sie „ausgewiesen“ bedeutet.

<u>Q4: In meiner Wohnumgebung wird den Radfahrer*innen genug Raum gegeben und die Radwege sind ausreichend breit.</u>

3.3) Wie muss Ihre Wohnumgebung aussehen, damit Radfahrer*innen „genug Raum“ haben?

3.4) Wie sehen für Sie „ausreichend breite“ Radwege aus?

<u>Q5: In meiner Wohnumgebung sind die Radwege in einem guten Zustand.</u>

3.5) Was bedeutet für Sie „guter Zustand“? Fallen Ihnen hierfür Synonyme ein?

3.6) Ihre Antwort bei dieser Frage war X. Wieso haben Sie sich für diese Antwort entschieden, und nicht für einen höheren oder niedrigeren Wert?

<u>Q7: In meiner Wohnumgebung kann ich Haltestellen des öffentlichen Nahverkehrs (Bus/Bahn) gut mit dem Fahrrad erreichen.</u>

3.7) Haben Sie in den letzten 2 Monaten mal das Fahrrad genutzt, um zu einer Haltestelle des öffentlichen Nahverkehrs zu gelangen, also zum Beispiel zum Bus oder zum Zug oder zur S-Bahn?

[WENN JA] 3.8) Würden Sie sagen, dass Ihre Wohnumgebung besser oder schlechter dafür geeignet ist, den öffentlichen Nahverkehr mit dem Fahrrad zu erreichen und wieso?

3.9) Was hat diese Frage in Ihnen hervorgerufen?

Q9: In meiner Wohnumgebung gibt es viele sichere Fahrrad-Abstellanlagen.

3.10) Beschreiben Sie, wie für Sie Fahrrad-Abstellanlagen aussehen müssen, damit diese als „sicher“ zu bezeichnen sind. Geben Sie gern Beispiele für „sichere“ Abstellanlagen.

3.11) Wie sind Sie bei der Beantwortung dieser Frage vorgegangen, um sich für eine Antwort zu entscheiden? Beschreiben Sie gern.

3.12) Wie sicher sind Sie sich, dass die Fahrrad-Abstellanlagen in Ihrer Wohnumgebung sicher sind?

Q10: In meiner Wohnumgebung fühle ich mich aufgrund der Verkehrssituation beim Radfahren sicher.

3.13) Bitte beschreiben Sie, wie Ihre Wohnumgebung aussehen muss, damit Sie dort sicher im Verkehr mit dem Fahrrad unterwegs sein können.

Q11: Meine Wohnumgebung ist eine schöne Umgebung um Fahrrad zu fahren.

3.14) Was bedeutet für Sie eine „schöne Umgebung“ zum Fahrradfahren?

3.15) Wie würden Sie diese Frage in eigenen Worten ausdrücken?

3.16) Finden Sie, dass diese Frage zu persönlich ist oder denken Sie, dass es in Ordnung ist, eine solche Frage in einer Umfrage gestellt zu bekommen?

Q13: In meiner Wohnumgebung fahren viele Menschen mit dem Fahrrad.

3.17) Wie definieren Sie hier „viel“?

3.18) Wie leicht oder schwer ist es Ihnen gefallen diese Frage zu beantworten?

Q14: Ich bewerte meine Wohnumgebung insgesamt als radfahrfreundlich.

3.19) Wie leicht oder schwer ist es Ihnen gefallen, diese Frage zu beantworten? (Falls eher schwer: Warum fanden Sie die Beantwortung dieser Frage eher schwer?)

Abschließend haben wir noch ein paar allgemeine Fragen, die sich auf beide Fragebögen, also den Walkability-Fragebogen und den Bikeability-Fragebogen, beziehen.

4) Allgemeine Fragen zum Walkability- und Bikeability-Fragebogen

4.1) Wenn Sie an beide Fragebögen denken, welchen fanden Sie einfacher zu beantworten und warum? Nennen Sie gern Beispiele.

4.2) Beschreiben Sie bitte einmal, wie Sie bei der Beantwortung der Fragen vorgegangen sind.

4.3) Fanden Sie die Anweisung zum Ausfüllen des Fragebogens verständlich oder unverständlich?

4.4) In der ersten Frage der beiden Fragebögen steht, dass Sie an Ihre „unmittelbare Wohnumgebung“ denken sollen, die „in 10 – 15 Minuten zu Fuß oder mit dem Fahrrad zu erreichen“ ist. Was verstehen Sie unter „10 – 15 Minuten um Ihren Wohnsitz“? Beschreiben Sie bitte, welche Orte oder Entfernungen Sie damit verbinden, aus Fußgänger*innen- und Radfahrperspektive, und wie Sie sich diese vorstellen.

4.5) Fanden Sie die Auswahlmöglichkeiten (von „Stimme überhaupt nicht zu“ bis „Stimme vollständig zu“) passend oder unpassend für die Beantwortung der Fragen?

4.6) Fanden Sie es einfach oder schwer, sich an Ihre Wohnumgebung zu erinnern? Wie haben Sie sich daran erinnert? Geben Sie gern Beispiele.

4.7) Erinnern Sie sich spontan an eine Frage, bei der Sie es besonders schwierig fanden, eine Antwort zu geben? Wieso war die Beantwortung dieser Frage schwer?

4.8) Hat Ihnen etwas im Fragebogen gefehlt, wenn Sie an Fußgänger*innenfreundlichkeit oder Radfahrfreundlichkeit in Ihrer Wohnumgebung denken? Wenn ja, was?

4.9) Sind Sie sich sicher oder unsicher, dass Ihre Antworten auf die Fragen zutreffend sind?

4.10) Menschen reagieren auf Fragen teilweise sehr unterschiedlich. Wir würden gerne Ihre Meinung zu diesen Fragen wissen. Denken Sie, dass diese Fragen für die befragte Person unangenehm sind oder eher nicht?

Ich möchte an dieser Stelle auch noch einmal daran erinnern, dass Ihre Daten streng vertraulich behandelt werden und dass Ihre Teilnahme an diesem Interview bedeutend zur Verbesserung des Fragebogens beiträgt. Wir wissen das sehr zu schätzen. Haben Sie noch Fragen oder Anmerkungen zu den beiden Fragebögen, unserem Interview oder unserer Studie allgemein?

Wir sind nun fertig mit unserem Interview. Vielen Dank noch einmal für Ihre Teilnahme an unserer Studie. Ich werde die Aufnahme nun beenden.

[TONAUFNAHME BEENDEN]

Einen schönen Tag wünsche ich noch, auf Wiedersehen.

[ENDE]
