# Supplementary material for: Development of survey instruments for assessing walkability and bikeability for the prevention indicator system of the German federal states
Source: Bundesgesundheitsblatt Gesundheitsforschung Gesundheitsschutz. 2025 Dec 2;69(1):51–60. [Article in German] doi: 10.1007/s00103-025-04163-w (PMC12764655; doi:10.1007/s00103-025-04163-w)
Supplement: Supplementary file 3 — Onlinematerial 3: Walkability [file 103_2025_4163_MOESM3_ESM.pdf]

Walkability

<u>Zu Fuß gehen in Ihrer Wohnumgebung</u>

Mit den nachfolgenden Fragen möchten wir erfahren, wie fußgängerfreundlich Ihre unmittelbare Wohnumgebung ist.

Denken Sie nun an Ihre unmittelbare Wohnumgebung um Ihren Wohnsitz, die zu Fuß in 10 - 15 Minuten zu erreichen ist. Bitte markieren Sie zu den unten aufgeführten Aussagen jeweils die aus Ihrer Sicht zutreffendste Antwort.

	**Stimme überhaupt nicht zu**	**Stimme eher nicht zu**	**Stimme eher zu**	**Stimme Vollständig zu**	**Weiß nicht/keine Angabe**
1. In meiner Wohnumgebung gibt es viele Einrichtungen des täglichen Bedarfs wie Geschäfte, Restaurants, Apotheken, Freizeiteinrichtungen und Schulen, die innerhalb von 10 - 15 min zu Fuß zu erreichen sind.	☐	☐	☐	☐	☐
2. In meiner Wohnumgebung gibt es eine gut ausgebaute Gehweginfrastruktur.	☐	☐	☐	☐	☐
3. In meiner Wohnumgebung wird den Fußgänger:innen genug Raum gegeben und die Gehwege sind ausreichend breit.	☐	☐	☐	☐	☐
4. In meiner Wohnumgebung sind die Gehwege in einem guten Zustand.	☐	☐	☐	☐	☐
5. In meiner Wohnumgebung kann ich Haltestellen des öffentlichen Nahverkehrs (Bus/Bahn) gut zu Fuß erreichen.	☐	☐	☐	☐	☐
6. In meiner Wohnumgebung fühle ich mich beim zu Fuß gehen vor Kriminalität sicher.	☐	☐	☐	☐	☐
7. In meiner Wohnumgebung gibt es viele verkehrsberuhigte Abschnitte wie 30er Zonen, Spielstraßen oder Fußgängerzonen.	☐	☐	☐	☐	☐
8. In meiner Wohnumgebung gibt es viele Zebrastreifen, Fußgängerampeln, Brücken oder Unterführungen.	☐	☐	☐	☐	☐
9. In meiner Wohnumgebung fühle ich mich aufgrund der Verkehrssituation beim zu Fuß gehen sicher.	☐	☐	☐	☐	☐
10. Meine Wohnumgebung ist eine schöne Umgebung um zu Fuß zu gehen.	☐	☐	☐	☐	☐
11. In meiner Wohnumgebung sind die Gehwege barrierearm, beispielsweise gibt es abgesenkte Bordsteine, Bodenleitsysteme oder Ampeln mit Tonsignal.	☐	☐	☐	☐	☐

12. In meiner Wohnumgebung gehen viele Menschen zu Fuß.	☐	☐	☐	☐	☐
13. Ich bewerte meine Wohnumgebung insgesamt als fußgängerfreundlich.	☐	☐	☐	☐	☐
