# Supplementary material for: Development of survey instruments for assessing walkability and bikeability for the prevention indicator system of the German federal states
Source: Bundesgesundheitsblatt Gesundheitsforschung Gesundheitsschutz. 2025 Dec 2;69(1):51–60. [Article in German] doi: 10.1007/s00103-025-04163-w (PMC12764655; doi:10.1007/s00103-025-04163-w)
Supplement: Supplementary file 4 — Onlinematerial 4: Bikeability [file 103_2025_4163_MOESM4_ESM.pdf]

Bikeability

Fahrrad fahren in Ihrer Wohnumgebung

Mit den nachfolgenden Fragen möchten wir erfahren, wie fahrradfreundlich Ihre unmittelbare Wohnumgebung ist. Denken Sie nun an Ihre unmittelbare Wohnumgebung um Ihren Wohnsitz, die mit dem Fahrrad in 10 - 15 Minuten zu erreichen ist. Bitte markieren Sie zu den unten aufgeführten Aussagen jeweils die aus Ihrer Sicht zutreffendste Antwort.

	**Stimme überhaupt nicht zu**	**Stimme eher nicht zu**	**Stimme eher zu**	**Stimme vollständig zu**	**Weiß nicht/keine Angabe**
1. In meiner Wohnumgebung gibt es viele Einrichtungen des täglichen Bedarfs wie Geschäfte, Restaurants, Apotheken, Freizeiteinrichtungen und Schulen, die innerhalb von 10 - 15 min mit dem Fahrrad zu erreichen sind.	☐	☐	☐	☐	☐
2. In meiner Wohnumgebung gibt es viele ausgewiesene Radstreifen und Radwege.	☐	☐	☐	☐	☐
3. In meiner Wohnumgebung gibt es viele ausgewiesene Radschnellwege und Fahrradstraßen.	☐	☐	☐	☐	☐
4. In meiner Wohnumgebung wird den Radfahrer:innen genug Raum gegeben und die Radwege sind ausreichend breit.	☐	☐	☐	☐	☐
5. In meiner Wohnumgebung sind die Radwege in einem guten Zustand.	☐	☐	☐	☐	☐
6. In meiner Wohnumgebung gibt es Radwege, die vom Verkehr getrennt sind.	☐	☐	☐	☐	☐
7. In meiner Wohnumgebung kann ich Haltestellen des öffentlichen Nahverkehrs (Bus/Bahn) gut mit dem Fahrrad erreichen.	☐	☐	☐	☐	☐
8. Der öffentliche Nahverkehr (Bus/Bahn) in meiner Wohnumgebung bietet mir Gelegenheit, mein Fahrrad mitzunehmen.	☐	☐	☐	☐	☐
9. In meiner Wohnumgebung gibt es viele sichere Fahrrad-Abstellanlagen.	☐	☐	☐	☐	☐
10. In meiner Wohnumgebung fühle ich mich aufgrund der Verkehrssituation beim Radfahren sicher.	☐	☐	☐	☐	☐
11. Meine Wohnumgebung ist eine schöne Umgebung um Fahrrad zu fahren.	☐	☐	☐	☐	☐

12. In meiner Wohnumgebung sind die Ampeln so geschaltet, dass ich als Radfahrer:in zügig vorankomme.	☐	☐	☐	☐	☐
13. In meiner Wohnumgebung fahren viele Menschen mit dem Fahrrad.	☐	☐	☐	☐	☐
14. Ich bewerte meine Wohnumgebung insgesamt als fahrradfreundlich.	☐	☐	☐	☐	☐
